# Successful Treatment of Anti-N-Methyl-D-Aspartate Receptor Encephalitis With Bilateral Ovarian Teratomas Through Three Surgeries Without Loss of Fertility

**DOI:** 10.7759/cureus.81381

**Published:** 2025-03-28

**Authors:** Kazuma Iwata, Daisuke Hamaguchi, Takamitsu Mizota, Yuki Matsuoka, Kiyonori Miura

**Affiliations:** 1 Department of Obstetrics and Gynecology, Nagasaki University Graduate School of Biomedical Sciences, Nagasaki, JPN; 2 Department of Obstetrics and Gynecology, Isahaya General Hospital, Isahaya, JPN; 3 Department of Neurology, Isahaya General Hospital, Isahaya, JPN; 4 Department of Pathology, Nagasaki University Hospital, Nagasaki, JPN

**Keywords:** anti-n-methyl-d-aspartate receptor encephalitis, fertility preservation, laparoscopic gynecological surgery, mature cystic ovarian teratoma, recurrent ovarian tumor

## Abstract

Anti-N-methyl-D-aspartate (NMDA) receptor encephalitis is associated with ovarian teratomas in approximately half of all cases. Surgical removal of these teratomas, combined with immunotherapy, results in rapid improvement in about half of patients. However, the remaining patients exhibit slower improvement and are at risk of severe complications. Additional surgeries may be considered for these patients. Since even microscopic teratomas can contribute to treatment resistance, complete removal of the remaining ovaries is often selected as a surgical approach. However, this approach results in loss of fertility. We report the case of a 28-year-old woman with bilateral ovarian teratomas and a refractory clinical course after initial treatment, including left salpingo-oophorectomy and right cystectomy. She underwent two additional surgeries, one for a residual teratoma and the other for a recurrent teratoma, both involving cystectomies aimed at preserving fertility. She was discharged home a year after admission and gave birth to a baby two years later. Our case is unique in that three surgeries were performed for an anti-NMDA receptor encephalitis patient with teratomas without loss of fertility, and it documents the reproductive outcome of the patient.

## Introduction

Anti-N-methyl-D-aspartate (NMDA) receptor encephalitis is an autoimmune disease caused by anti-NMDA receptor autoantibodies. The NMDA receptor is a transmembrane ligand-gated cation channel widely distributed in neuronal cells of the central nervous system and plays a key role in synaptic plasticity and memory formation. Autoantibodies binding to NR1 subunits of NMDA receptors cause receptor internalization, reducing their cell surface numbers [1]. The resulting clinical symptoms are similar to those caused by NMDA receptor antagonists such as ketamine and nitrous oxide, as well as to schizophrenia, where NMDA receptor hypofunction occurs. In the typical presentation, a week of nonspecific viral-like symptoms such as high fever and headache is followed by rapidly progressive cognitive impairment that manifests as delusions, hallucinations, agitation, and disorganized speech. Neurological complications ensue, including seizures and central hypoventilation [2], which may require intensive treatment with deep sedation and mechanical ventilation. The mortality rate is 5-7%, and the causes of death include severe pneumonia, multiorgan failure, and refractory status epilepticus [3].

Anti-NMDA receptor encephalitis is now well known to be associated with ovarian teratomas in approximately half of all cases. Ovarian teratomas are the most common type of germ cell tumor of the ovary, accounting for 95% of cases. These tumors consist of well-differentiated tissues derived from at least two of the three germ layers: ectoderm, mesoderm, and endoderm. In 88% of cases, teratomas are filled with sebaceous material, a highly specific feature that serves as a hallmark for their radiographic diagnosis. Skin and skin appendages are present in nearly all cases, whereas nervous tissues, which share the same embryonic dermal origin as skin and skin appendages, are less frequent, occurring in 32.3% of cases [4]. Chefdeville et al. reported a significantly higher frequency of nervous tissues (96%) in teratomas associated with anti-NMDA receptor encephalitis compared to those without (38%), suggesting a potential role for neural tissues in the pathogenesis of this disease [5].

In 2016, Graus et al. [6] proposed the diagnostic criteria for anti-NMDA receptor encephalitis. According to the diagnostic criteria, in the presence of a systemic teratoma, the diagnosis of probable anti-NMDA receptor encephalitis can be established based solely on the patient's symptoms, which may expedite the initiation of treatment. Treatment consists of immunotherapy and surgical removal of a teratoma if present. Immunotherapy includes IV methylprednisolone, IV immunoglobulin, and plasma exchange as first-line immunotherapy and rituximab and cyclophosphamide as second-line immunotherapy. For those who do not improve even with second-line immunotherapies, biologics and intrathecal administration of methotrexate or rituximab have been reported, although the evidence of their use is still limited. Regarding surgical treatment, although a systematic review fails to demonstrate robust evidence about the effectiveness and appropriate timing of surgery [7], there are numerous case reports and case series where surgical removal, in combination with or even without immunotherapies, leads to clinical improvements. The pediatric guideline for anti-NMDA receptor encephalitis, which is the only guideline available for the disease, states that tumor removal is required as it can result in rapid improvements [8]. Based on these literature and expert opinions, surgical removal of a teratoma is considered standard if it is recognized in imaging studies.

The first-line immunotherapies, plus tumor removal if applicable, result in rapid improvement, which is defined as a modified Rankin Scale (mRS) ≤2 within four weeks in nearly half of cases. Meanwhile, the rest tends to improve more slowly on a yearly basis even if they were treated with the second-line immunotherapy. In one year after onset of disease, 40% still needed some assistance in daily life (mRS>3), and half of them continued to do so even two years after onset of disease [9]. Considering the poor prognosis of refractory cases, many cases undergo an additional operation to remove all remaining ovaries alongside second-line immunotherapies [10-13], as a microscopic teratoma, detectable only through pathological inspection, can be associated with anti-NMDA receptor encephalitis [14,15]. However, this approach results in loss of fertility. Fertility concerns in young women with anti-NMDA receptor encephalitis and ovarian teratomas are significant, particularly when the teratomas are bilateral or when the clinical course is refractory, necessitating multiple surgeries. 

We report a refractory case of anti-NMDA receptor encephalitis with bilateral teratomas undergoing three surgical operations. She finally improved while sparing the right ovary and delivered a term baby three years after the onset of the disease. As far as we know, this is the first report of a baby being delivered by an anti-NMDA receptor encephalitis patient with a refractory clinical course who underwent multiple surgeries.

## Case presentation

**Figure 1:**
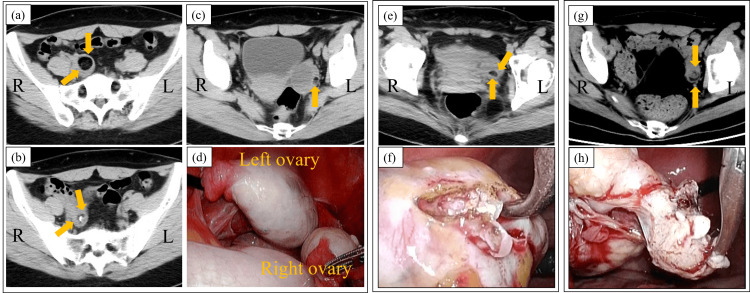
CT and surgical findings during the clinical course. (a-c) Pelvic CT performed at admission revealed bilateral ovarian cysts with fatty components (yellow arrows): two in the right ovary and one in the left, suggesting the presence of bilateral teratomas. One of the cysts also contains calcification, a common finding in teratomas. (d) On day 3, right salpingo-oophorectomy and left ovarian cystectomy were performed. During the procedure, a single large cyst in the left ovary was clearly identified. However, the cysts intended for removal could not be located in the right ovary. Pathological examination confirmed the presence of mature teratomas in the right ovary, while the left cyst was identified as a luteinized follicle. (e) A pelvic CT performed on day 43 confirmed a cyst with a fatty component (yellow arrows), which was the same in size and location as the CT taken at admission. (f) The second surgery was conducted to remove the residual teratoma. During the operation, the teratoma was recognizable by its yellowish appearance on the ovarian surface. Removal of the whole ovary was avoided to preserve fertility. Pathological examination confirmed the correct resection of the tumor. (g) Pelvic CT on day 191 found an ovarian cyst with a fatty component (yellow arrows). Recurrent teratoma was diagnosed because the previous CT showed no cyst. (h) In the third operation, the teratoma containing hair was successfully removed.

A 28-year-old woman with no significant past medical history presented to our hospital with altered mental status. She was married and nulliparous. Her husband stated that she began experiencing altered mental status a day prior to admission, preceded by a severe headache persisting for a week. On presentation, she exhibited disorientation, disorganized speech, hallucinations, and agitation. She was febrile, with a body temperature of 38.5℃, and tachycardic, with a heart rate of 136 bpm. Her blood pressure was 137/95 mmHg, and her oxygen saturation was 99% on room air. Neurological and physical examinations were normal, except for the presence of orofacial dyskinesia. Blood tests revealed no abnormalities. Cerebrospinal fluid (CSF) analysis showed a slightly elevated opening pressure of 245 mmH_2_O and an increased white blood cell count (Table 1). Brain MRI revealed high signal intensity on FLAIR imaging along the inner surface of the temporal lobes bilaterally. A whole-body CT scan showed three ovarian cysts with fatty components, two on the right ovary and one on the left, consistent with a diagnosis of teratoma (Fig. 1a-c). Based on the CT findings and her clinical presentation, a diagnosis of probable anti-NMDA receptor encephalitis was made. CSF and serum samples were sent to Niigata University for anti-NMDA antibody testing, which later confirmed the presence of antibodies in both serum and CSF, establishing a definitive diagnosis of anti-NMDA receptor encephalitis.

**Table 1:** CSF analysis on admission (day 1). Cryptococcal antigen, herpes simplex virus antibodies (IgM and IgG), and HSV DNA (HSV1 and HSV2) were all negative in the CSF. Additionally, autoimmune encephalitis-associated cell surface antibodies, including antibodies against LG1, CASPR2, AMPAR, GABA_B_R, and DPPX, were all negative, except for NMDA receptor antibodies. CSF: cerebrospinal fluid; NMDA: N-methyl-D-aspartate; HSV: herpes simplex virus.

CSF analysis		
Parameter	Result	Reference range
Appearance	Clear	Clear
Pressure	245 mmH_2_O	50–200 mmH_2_O
White blood cell count	380/μL	≤5/μL
Mononuclear	370/μL	
Polynuclear	10/μL	
Protein	45 mg/dL	15–45 mg/dL
Glucose	58 mg/dL	50–80 mg/dL
Microbial culture	Negative	Negative
NMDA receptor antibody	Positive	Negative

She was hospitalized on the day of presentation (day 1), and prompt initial therapies, including first-line immunotherapy and surgical removal of bilateral teratomas, were planned. Soon after admission, ventilator management was initiated due to the onset of central hypoventilation. On day 3, right salpingo-oophorectomy and left ovarian cystectomy were performed (Fig. 1d). During the procedure, a single large cyst in the left ovary was easily identified and enucleated. However, the cysts intended to be removed could not be located in the right ovary due to the presence of multiple surface cysts, necessitating a salpingo-oophorectomy. Postoperatively, the patient remained intubated due to persistent agitation and recurrent seizures since admission. The seizures involved tonic activity in the limbs, accompanied by upward deviation of the eyes, which could not be fully controlled despite the administration of propofol and midazolam. On day 5, intravenous immunoglobulin (25 g/day) and methylprednisolone (1000 mg/day) were administered for five days. Despite these interventions, her condition did not improve. She became unresponsive to physical stimuli and experienced persistent hypoventilation with intermittent apnea, which required continued intubation. A tracheostomy was performed on day 21. Over the next two weeks, seizure frequency decreased, but her level of consciousness remained unchanged.

As a second-line therapy, weekly rituximab (600 mg, once weekly for four doses) was initiated on day 35, but no clinical improvement was observed. A CT scan on day 60 revealed a teratoma in the left ovary (Fig. 1e). It was considered a residual rather than a recurrent teratoma, as the cyst enucleated during the first surgery was identified as a luteinized follicle and showed no evidence of teratoma. Given her persistent unconsciousness despite rituximab treatment, a second surgery was performed on day 81 to remove the residual teratoma (Fig. 1f). Although complete removal of the left ovary could have been an option, it was decided to perform a cystectomy after careful discussions with her husband and family, who wanted to preserve her fertility if possible. Pathological analysis of the enucleated cyst confirmed successful resection of the teratoma.

Shortly after the second surgery, the patient began exhibiting pursuit eye movements and voluntary movements. She was extubated on day 107, three weeks postoperatively. However, with no further significant improvement, methylprednisolone (1000 mg/week for three doses) was administered starting on day 143, followed by a single dose of cyclophosphamide (1000 mg) on day 172. On day 176, for the first time since her initial surgery, the patient communicated with medical staff using simple words. Subsequently, she demonstrated increased awareness of her surroundings and began engaging more actively, using complex language. On day 187, methotrexate (8 mg/week, orally) was initiated as maintenance therapy for a duration of one year. Although a CT scan on day 191 revealed a small cyst in the left ovary, highly suspicious for recurrent teratoma (Fig. 1g), surgical intervention was initially withheld as the patient continued to show clinical improvement.

**Figure 2:**
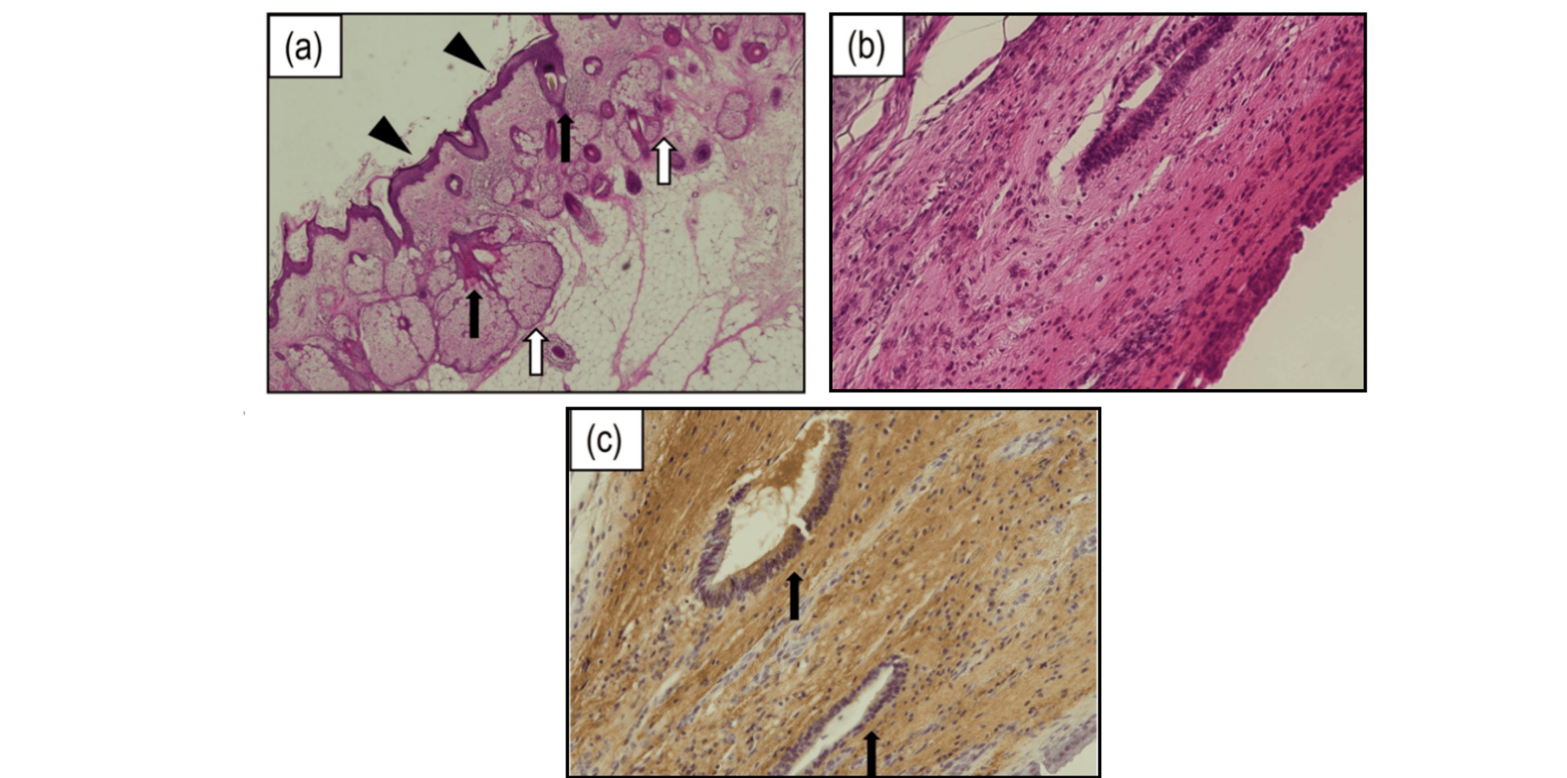
Photomicrograph of the enucleated cyst in the third operation. (a) Normal dermis structure consists of squamous cell epithelium (black arrowheads), hair follicles (black arrow), and sebaceous glands (white arrows). This picture demonstrates typical histological features commonly found in teratomas (H&E ×40). (b) Mature glial tissue, as well as peripheral nerve tissue (not shown here), was present (H&E ×40). (c) Tubal structures lined with columnar epithelial cells (black arrows) were recognized among mature glial tissue. The epithelial cells had nuclei with mild atypia and weak overlapping and were positive for S100 and GFAP, demonstrating that they were neural tubes. The presence of nervous tissues in our case aligns with the findings of Chefdeville et al., who reported that nervous tissues are present in nearly all cases of teratomas associated with anti-NMDA receptor encephalitis. H&E: hematoxylin and eosin; NMDA: N-methyl-D-aspartate.

**Figure 3:**
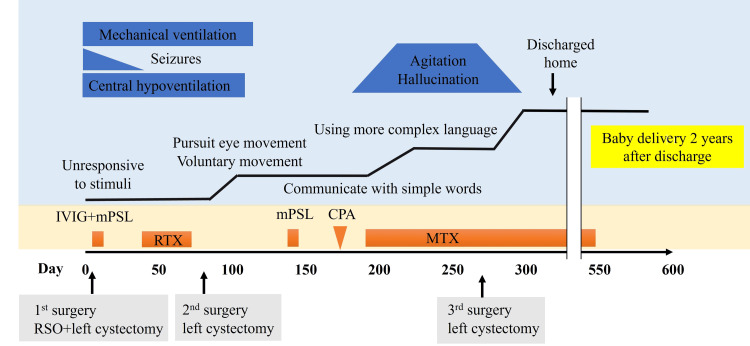
Clinical course of our patient. IVIG+mPSL: intravenous immunoglobulin (25 g/day) and methylprednisolone (1000 mg/day) for five days; RTX: weekly rituximab (600 mg once a week, four times in total); mPSL: methylprednisolone (1000 mg once a week, three times in total); CPA: a single dose of cyclophosphamide (1000 mg); MTX: methotrexate (8 mg/week for a year).

Meanwhile, as her level of consciousness improved, neuropsychiatric symptoms such as irritability and hallucinations became apparent. As these symptoms persisted, a left cystectomy was performed on day 269 (Fig. 1g). Pathology was consistent with teratoma (Fig. 2). She was transferred to a rehabilitation hospital on day 278 and discharged home two months later when her neuropsychiatric symptoms subsided. One year after discharge, the patient had a spontaneous pregnancy. The course of her pregnancy progressed well, and she delivered a healthy baby at term by emergency cesarean section due to prolonged decelerations during delivery. The prolonged decelerations were attributed to umbilical compression, as no other contributing factors were identified. She is currently under follow-up with no signs of recurrence. She has returned to work and exhibits no apparent sequelae. Figure 3 shows the clinical course of the patient. Informed consent was obtained from the patient to publish this case report, and patient anonymity was preserved.

## Discussion

This case demonstrates the successful reproductive outcome of a patient with anti-NMDA receptor encephalitis and bilateral teratomas, who underwent three surgeries during her refractory clinical course. Despite the complete removal of the right ovary and three cystectomies on the left ovary, her fertility was preserved, as demonstrated by the successful delivery of her baby.

Our case report is unique in two aspects. First, no existing literature documents as many as three surgical procedures performed for an anti-NMDA receptor encephalitis patient with teratomas. Second, this is the first report to detail the reproductive outcome of a patient with anti-NMDA receptor encephalitis who underwent multiple surgeries. A two-year follow-up after discharge confirmed the successful preservation of her fertility, as evidenced by the delivery of her baby.

To our knowledge, six cases undergoing additional surgeries for refractory or recurrent clinical courses have been reported and are summarized in Table 2 [10-13,16]. Although all patients were of childbearing age, ranging from 13 to 27, all remaining ovaries were removed during the second surgery in five cases. In four of these five cases, second surgeries were performed despite no radiological evidence of recurrent or residual teratomas on preoperative imaging. The rationale behind this approach is that both microscopic and radiologically apparent teratomas are associated with anti-NMDA receptor encephalitis, contributing to treatment resistance. For patients suffering from intractable recurrent seizures or life-threatening central hypoventilation, bilateral ovary removal may be necessary, regardless of the presence of radiologically apparent teratomas in the ovaries.

**Table 2:** Refractory or recurrent cases of anti-NMDA receptor encephalitis undergoing second surgery. NMDA: N-methyl-D-aspartate; RSO: right salpingo-oophorectomy; LSO: left salpingo-oophorectomy; BSO: bilateral salpingo-oophorectomy; LO: left oophorectomy. For "cystectomy," the letters preceding it (L, B, R) indicate left, bilateral, and right, respectively. ^1^Preoperative state indicates preoperative state before second surgery. ^2^Macroscopic tumor indicates whether a macroscopic tumor suspected to be a teratoma is present based on imaging studies. ^3^Postoperative course indicates postoperative course after second surgery.

Reference	Age	First surgery	Preoperative state^1^	Macroscopic tumor^2^	Second surgery	Postoperative course^3^	Third surgery	Ovary preserved	Delivery documented
Our case	28	RSO + L cystectomy	Unresponsive to stimuli	Left side	L cystectomy	Improving	L cystectomy	Yes	Yes
Dabner et al. [16]	27	RSO	Catatonic	Left side	Partial LO	Improving	No	Yes	No
Cirkel et al. [10]	21	R cystectomy	Status epilepticus, permanently sedated	No	BSO	Refractory	No	No	No
Chiriboga et al. [11]	13	LSO	Status epilepticus	No	RSO	Improving	No	No	No
Uchida et al. [12]	20	B cystectomy	Coma	Left side	BSO	Improved	No	No	No
Saeed et al. [13]	23	RSO + partial LO	Seizures, behavioral abnormalities	No	LSO	Improved	No	No	No
Saeed et al. [13]	27	L cystectomy	Status epilepticus, coma	No	BSO	Refractory	No	No	No

Meanwhile, Iizuka et al. described three patients with anti-NMDA receptor encephalitis and teratomas who recovered without tumor removal, albeit slowly [17]. Shindo et al. reported two similar cases [18]. Since not all patients with anti-NMDA receptor encephalitis require surgical removal of teratomas for recovery, watchful waiting or leaving some ovaries intact can be considered when patients are not in immediate danger. This strategy does carry risks: (1) Slower recoveries pose greater risks of complications, such as severe pneumonia, contracture, and even death. (2) Some cases seem to require teratoma removal for improvement [14,15]. (3) Mortality rate and neurological outcome are worse compared to tumor removal groups. Therefore, careful discussions with neurologists and patient families are prerequisite for this strategy. Moreover, flexibility is essential to allow for the surgical removal of all remaining ovarian tissue based on the clinical course, even if the initial approach was to opt for watchful waiting or preserve some ovarian tissue.

In our case, seizures were no longer observed after the initial treatment. Her respiratory condition remained stable, although she continued to require mechanical ventilation. This relatively stable condition allowed us to opt for a right cystectomy as the second surgery, respecting her husband and parents' desire to preserve her fertility. As for the third surgery, we withheld it upon recognizing the recurrent right teratoma due to observed clinical improvement at that time. However, we adjusted our strategy and proceeded with the surgery due to her prolonged irritability and hallucinations. A neurologist, who served as the doctor in charge, a gynecologist, her husband, and her parents were involved in these decisions. After being informed of the risks associated with cystectomy (i.e., a higher risk of treatment failure necessitating additional surgery and an increased likelihood of relapse after recovery), her husband and parents requested the preservation of ovarian function, if feasible. The neurologist assessed feasibility based on the patient’s clinical course, while the gynecologist evaluated it from a surgical perspective.

Ovarian tissue cryopreservation is a fertility preservation procedure for patients undergoing treatments that may compromise ovarian function. While it is typically performed for non-ovarian cancer patients prior to chemotherapy, a case involving a patient with bilateral teratomas has been reported [19]. Before the second surgery, a consultation was held with a reproductive endocrinology specialist to evaluate the feasibility of tissue cryopreservation in our case. He expressed concerns about the potential contamination of preserved tissue with teratoma components and opposed the procedure under the circumstances at that time. If the patient’s clinical course had been more severe, ovarian tissue cryopreservation could have been considered more favorably.

Dai et al. reported a relapse rate of 14.6% in anti NMDA receptor encephalitis patients with ovarian teratomas undergoing surgical removal, during a median follow-up of 37.69 months [20]. Additionally, there are documented cases where clinical relapses coincide with the recurrence of teratomas. Careful follow-up including transvaginal ultrasound is mandatory for patients who have fully recovered from anti-NMDA receptor encephalitis.

Heine et al. reported that patients with anti-NMDA receptor encephalitis frequently experience persistent cognitive deficits during long-term follow-up [21]. At 2.3 years post-onset, moderate to severe impairments were observed in over 80% of patients. Even at 4.9 years, despite significant improvements in cognitive function, moderate to severe deficits persisted in two-thirds of the patients. An mRS score of ≤2 is widely recognized as a benchmark for defining a favorable treatment outcome in anti-NMDA receptor encephalitis, as in the well-cited study by Titulaer et al. [9]. However, it is important to note that these patients may continue to experience cognitive dysfunction. In our case, the patient exhibited periodic emotional instability following discharge, which was alleviated with hormonal contraceptives. At 19 months post-onset (seven months after discharge), she underwent evaluation using the Wechsler Adult Intelligence Scale (WAIS), a widely utilized psychological test for assessing intelligence in adults and older adolescents. Her overall IQ score was average (100); however, she demonstrated lower-than-average performance in working memory (85), while achieving above-average scores in processing speed (116) and perceptual reasoning (112). We were uncertain whether these findings were attributable to the sequelae of the disease. The emotional instability might have been due to premenstrual syndrome, while the lower-than-average performance in working memory could reflect inherent cognitive traits, given her above-average performance in other cognitive domains. Since returning to work, she has been doing well without significant issues, which suggests that she does not have moderate to severe cognitive deficits. However, careful follow-up of her cognitive functions with quantitative assessments using validated tests is necessary, and attention should be paid to whether she can manage her daily life as she did prior to the onset of the disease.

Our patient underwent three surgeries, all of which included left cystectomy. While it can be stated that her fertility was preserved despite the repeated cystectomies, determining the contributions of each surgery to her clinical improvement is challenging due to the inherent limitations of a single case report.

## Conclusions

Our case report stands out for two key reasons. Firstly, no existing literature has documented as many as three surgical procedures for the treatment of anti-NMDA receptor encephalitis with teratomas. Secondly, this is the first report to demonstrate a successful reproductive outcome in a patient with anti-NMDA receptor encephalitis who underwent multiple surgeries.

While refractory cases treated with multiple surgeries are documented in the literature, reproductive outcomes remain unaddressed. Our case demonstrates that fertility preservation is achievable, even in refractory cases of anti-NMDA receptor encephalitis with teratomas, provided that the timing and procedures of surgeries are carefully and flexibly planned.
